# Epibatidine Blocks Eye-Specific Segregation in Ferret Dorsal Lateral Geniculate Nucleus during Stage III Retinal Waves

**DOI:** 10.1371/journal.pone.0118783

**Published:** 2015-03-20

**Authors:** Zachary W. Davis, Chao Sun, Brittany Derieg, Barbara Chapman, Hwai-Jong Cheng

**Affiliations:** 1 Center for Neuroscience, University of California Davis, Davis, California, United States of America; 2 Department of Neurobiology, Physiology, and Behavior, University of California Davis, Davis, California, United States of America; 3 Department of Pathology and Laboratory Medicine, University of California Davis, Davis, California, United States of America; Virginia Tech Carilion Research Institute, UNITED STATES

## Abstract

The segregation and maintenance of eye-specific inputs in the dorsal lateral geniculate nucleus (dLGN) during early postnatal development requires the patterned spontaneous activity of retinal waves. In contrast to the development of the mouse, ferret eye-specific segregation is not complete at the start of stage III glutamatergic retinal waves, and the remaining overlap is limited to the C/C1 lamina of the dLGN. To investigate the role of patterned spontaneous activity in this late segregation, we disrupted retinal waves pharmacologically for 5 day windows from postnatal day (P) 10 to P25. Multi-electrode array recordings of the retina *in vitro* reveal that the cholinergic agonist epibatidine disrupts correlated retinal activity during stage III waves. Epibatidine also prevents the segregation of eye-specific inputs *in vivo* during that period. Our results reveal a novel role for cholinergic influence on stage III retinal waves as an instructive signal for the continued segregation of eye-specific inputs in the ferret dLGN.

## Introduction

The segregation of eye specific projections from retinal ganglion cells (RGCs) in the dorsal lateral geniculate nucleus (dLGN) is used as a model to study the formation and modification of neural circuits. Previous work on this system indicates that spontaneous correlated activity in the form of retinal waves instructs this segregation [1–4] which occurs through the large scale bursting of retinotopically aligned ganglion cells within one eye that is temporally offset from bursting activity in the opposing eye. When the correlated activity of RGCs during cholinergic stage II retinal waves is disrupted with the cholinergic agonist epibatidine the RGC inputs fail to segregate [1,3,5]. Multi-electrode array (MEA) recordings of RGCs during stage II waves reveal that epibatidine eliminates the local correlations between adjacent RGCs [6–8] and epibatidine has been shown to abolish retinal waves *in vivo* [9]. Without these intra-eye correlations from retinal waves, the random residual bursting after epibatidine exposure is believed to drive relatively increased correlations between the two eyes, resulting in the inability for the system to segregate between the eye specific inputs.

In the mouse, the eye-specific segregation of RGC inputs in the dLGN is complete by the end of stage II waves around postnatal day (P) 10 [10,11]. The cholinergic network of starburst amacrine cells (SACs) in the retina disassembles rapidly afterwards [12,13] and epibatidine treatment has no effect on locally correlated activity in the mouse retina during later stage III glutamatergic retinal waves [8,11]. However, this observation is not true in the ferret; epibatidine continues to effectively disrupt locally correlated activity during stage III retinal waves [8]. This persistence of the cholinergic influence on patterned spontaneous activity late in the ferret retina raises a specific issue as to the significance and necessity of the cholinergic network during stage III waves among higher mammals.

How does the persistence of the cholinergic network during stage III retinal waves affect the development of the ferret visual system? It has been shown that spontaneous activity during stage III waves in the ferret is necessary for the maintenance of eye-specific inputs, as activity blockade with high concentrations of the mGluR6 agonist 2-amino-4-phosphonobutyrate (APB) leads to a desegregation of inputs in the ferret dLGN [14]. However, eye-specific segregation is not complete at the start of stage III waves as overlap persists between the contralateral C and ipsilateral C1 lamina [15–17]; spontaneous activity is necessary for the segregation of these projections. The question remains as to the necessity of the persistent stage III cholinergic activity observed in the ferret during stage III glutamatergic retinal waves.

To explore the role of the cholinergic network in the segregation of RGC inputs during stage III waves in the ferret, we investigated whether cholinergic disruption of retinal activity could desegregate the inputs similar to activity blockade, and whether it could prevent further refinement of eye-specific inputs. To achieve this we disrupted retinal waves for 5 day windows with intraocular injections of epibatidine beginning at P10, P15, and P20. We examined the efficacy of epibatidine to disrupt correlated activity with MEA recordings of the retina and related it to the progression of eye-specific segregation. We found that the A/A1 lamina segregates by P10 but the late overlap specific to the C/C1 lamina segregates from P10 to P20. Epibatidine treatment prevents segregation after P10, but does not reverse it. From this we conclude that there are different epochs of eye-specific segregation for different laminar structures in the ferret, and that the correlated activity generated by the cholinergic network is necessary for, and persistent until, the completion of eye-specific segregation.

## Results

### Eye-specific Segregation in the Ferret dLGN Continues through Stage III Retinal Waves

**Fig 1:**
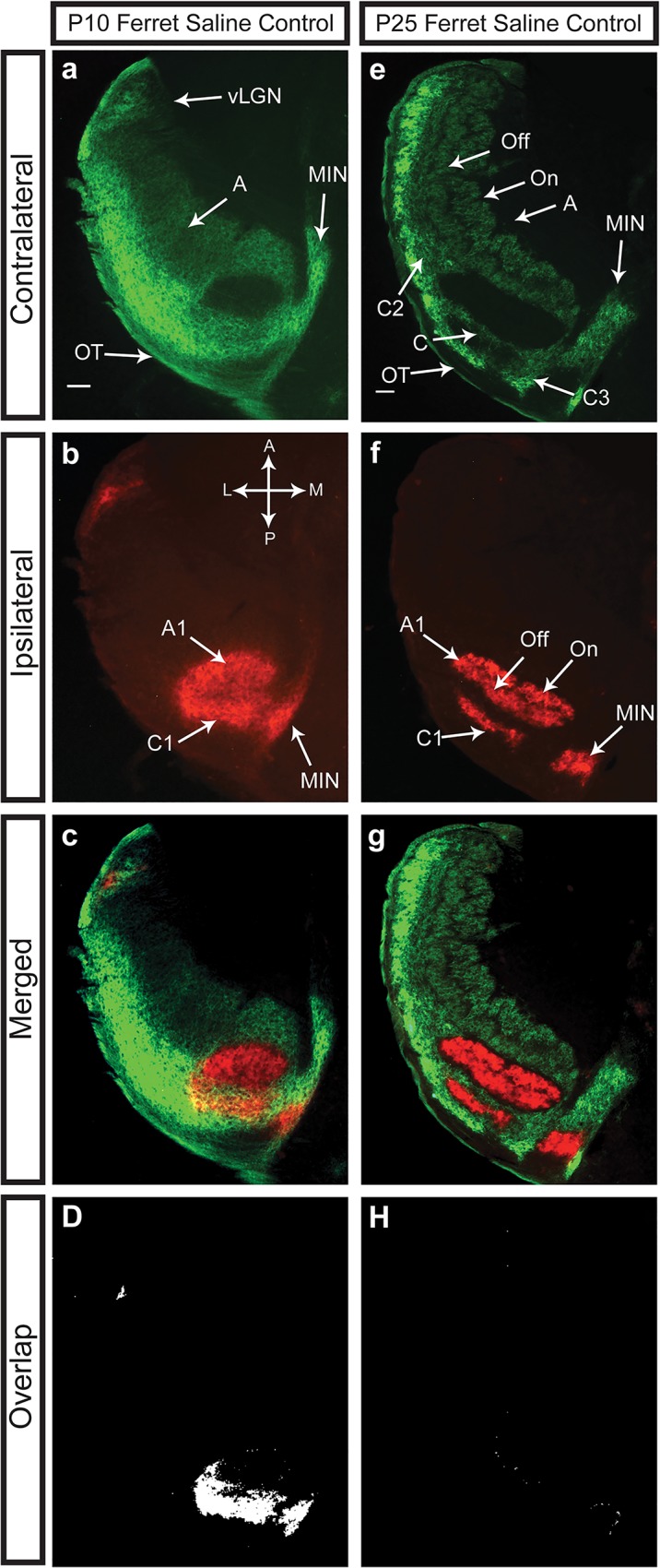
Eye-specific segregation is not complete at P10 in the ferret, but is by P25. Tissue is from representative sections sliced horizontally through the juvenile ferret dLGN. At P10 (A-D) the segregation of the retinal inputs from the left eye (green) and the right eye (red) in the A/A1 lamina is nearly complete with very little residual overlap remaining. In contrast, the C/C1 lamina is almost entirely overlapped between the two eyes. By P25 (E-H), both the A/A1 and C/C1 laminar regions have completely segregated leaving little to no overlap between either the A/A1 lamina or C/C1 lamina. OT, optic tract; A, contralateral A lamina; A1, ipsilateral A lamina; C, C2, C3, contralateral C lamina; C1, ipsilateral C lamina; On, Off, On and Off lamina; MIN, medial intralaminar nucleus; vLGN, ventral lateral geniculate nucleus. Scale bars are 200 μm.

The segregation of eye-specific inputs to the dLGN in the ferret is known to occur during the first three weeks of postnatal development [15–17]. In our control ferrets, the eye-specific inputs to the dLGN at P10 (*n* = 4) are not entirely segregated, but the total remaining area of overlap is relatively small [8.20 ± 1.31%, (SEM) at 30% above background threshold] (Fig. 1, A-D). This overlap is largely restricted to the C/C1 lamina, which are mostly overlapped at this age [69.90 ± 3.67%, (SEM)]. Consistent with previous work on the segregation of eye-specific inputs at P10, the A/A1 lamina is almost entirely segregated [3.39 ± 0.87%, (SEM)]. By P25 (*n* = 3) the remaining overlap observed at P10 is gone and the total overlap area is 0.67 ± 0.24% (SEM) of the total dLGN area, and 11.03 ± 4.13% (SEM) of the C/C1 lamina (Fig. 1, E-H). Therefore, the C/C1 lamina of the eye-specific inputs must segregate between P10 and P25 in the ferret, during which time the spontaneous activity of the retina is dominated by stage III glutamatergic retinal waves [18–21]. To explore the consequence of epibatidine treatment on the segregation and maintenance of eye-specific inputs after P10 in the ferret dLGN, we used (1) physiological MEA recordings to address the effects of epibatidine treatment on the retinal waves *in vitro* and (2) anatomical tracing to investigate the effects of epibatidine on late eye-specific segregation in the dLGN *in vivo*.

### Epibatidine Disrupts Correlated Activity During Glutamatergic Stage III Retinal Waves

**Fig 2:**
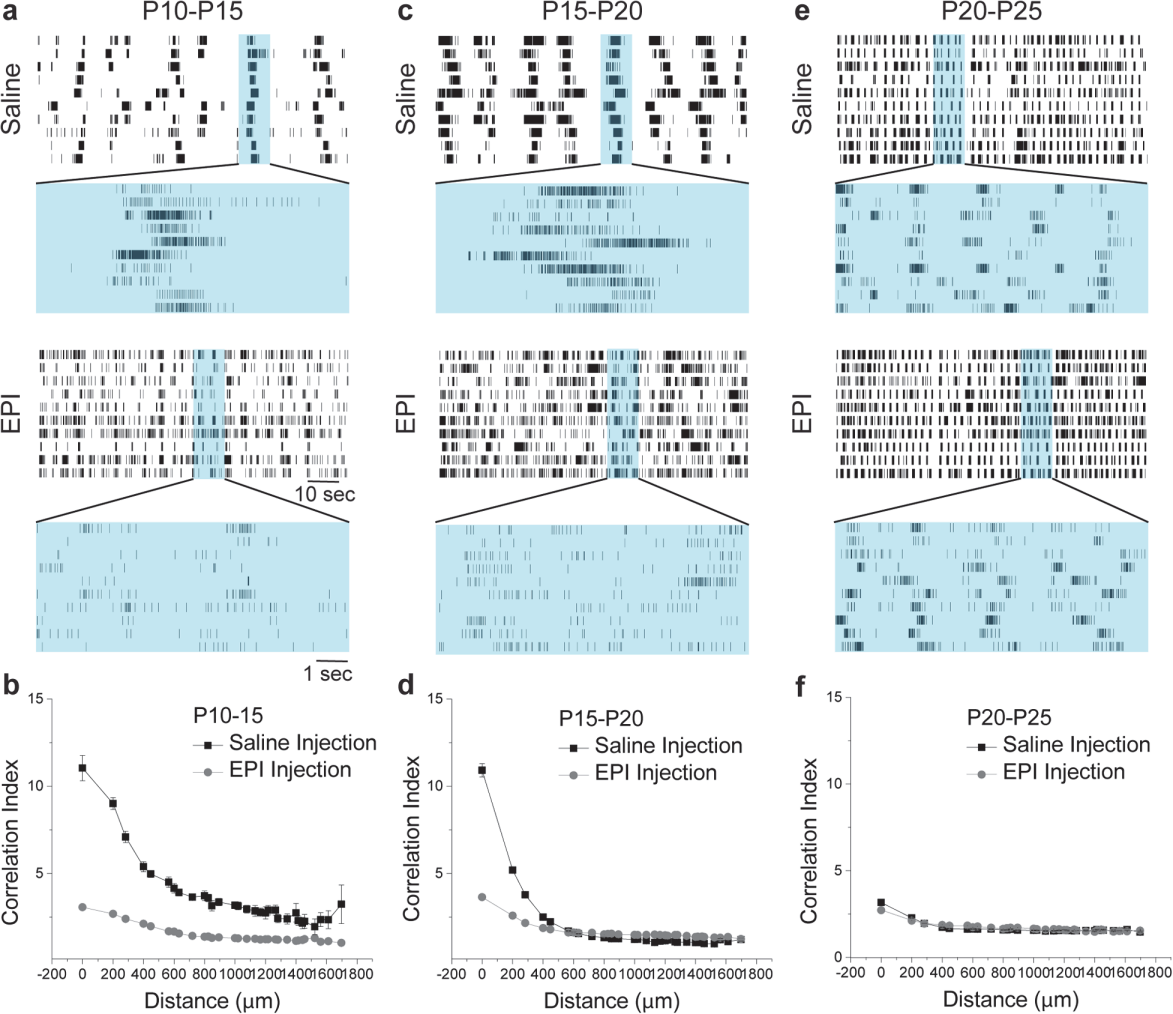
Correlated activity during stage III retinal waves is severely disrupted by epibatidine treatment. Ferrets treated with saline or epibatidine for 5 day windows were recorded on the multi-electrode array (MEA) in control bath solution at P15, P20, and P25. Normal stage III retinal waves are present in the spatially organized spike rasters of saline treated ferrets, but epibatidine treatment disrupts these patterns at P15 (A). Top raster for both saline and epibatidine treated ferrets displays global RGC firing during 2 minutes of a recording, while the bottom raster shows the spatial and temporal correlations present during a single wave event. The same result observed at P15 is present in the patterned activity of P20 ferrets (C) for both saline (top) and epibatidine treated (bottom) conditions. At P25 (E) there is no difference in the observed pattern of activity between saline (top) and epibatidine treated ferrets (bottom). The Correlation Index (CI) between pairs of cells decreases as a function of inter-pair distance in saline treated ferrets (B, D, and F, black dots). Epibatidine treatment from P10-P15 (B) and P15-P20 (D) reduces the correlated activity occurring during spontaneous retinal waves over short inter-pair distances (gray dots). At P25 there is a severe reduction in the correlated activity observed in saline treated ferrets, and their activity is no different in local correlations than epibatidine treated ferrets (F). Data points reflect mean values ± SEM.

Unlike previous reports in mice, epibatidine treatment results in a severe disruption in the patterned activity of ferret retinal ganglion cells at P15 and P20 (Fig. 2, A, and C). No obvious difference in the patterned activity is observed between saline treated and epibatidine treated ferrets at P25 (Fig. 2E). However, there is a clear difference in the patterns of normal stage III waves at P15 and P20 when compared to P25. This data indicates that the cholinergic network is influencing retinal waves up until P20, and the efficacy of epibatidine to disrupt the patterned spontaneous activity of retinal waves is attenuated or eliminated at some point between P20 and P25.

Correlated activity in retinal waves has been implicated as the instructive signal for eye-specific segregation [4,22–24]. To measure the correlated activity between cells during waves, we computed the correlation index (CI) [18], which conveys the likelihood relative to chance that a pair of cells at a given distance fired within the same window of time (see Materials and Methods). Larger values of the index indicate a greater likelihood of temporally correlated firing. We examined the spatial correlation of activities present during retinal waves *in vitro* in saline treated and epibatidine treated ferrets (Fig. 2, B, D, and F) by comparing pairs of units organized by their channel location on the MEA. At P15, the average CI between units separated 200μm is 11.01 ± 0.73 (SEM; *n* = 99 units from 2 retinas), which indicates a strong likelihood that adjacent cells are firing in a correlated fashion during our recordings. In comparison, epibatidine’s disruption of retinal waves eliminates the majority of these local correlations in the activity and reduces the CI to 3.07 ± 0.13 (SEM; *n* = 135 units from 2 retinas) (Fig. 2, B).

Similar results are observed in ferrets recorded at P20. The CI at 200μm is 10.92 ± 0.38 (SEM; *n* = 711 units from 6 retinas), but falls off much more sharply than at P15, indicating that correlations across distances of up to 1400μm at P15 were only found at distances of up to 400μm at P20 (Fig. 2, D). This may reflect a difference in the developmental process occurring at these time points; older ages undergo more fine scale retinotopic refinement that would be better instructed by correlated activity over smaller retinal distances[18,19]. Regardless of these intrinsic differences at P15 and P20, epibatidine continues to severely attenuate the local correlations at P20 [CI = 3.65 ± 0.10 (SEM) at 200μm; *n* = 483 units from 5 retinas].

A different pattern is observed at P25, both in the normal patterned activity as well as the consequence of epibatidine treatment. The normal spontaneous activity no longer shows the correlations across any distance of the retina; the average CI at 200μm is 3.17 ± 0.06 (SEM; *n* = 410 units from 4 retinas). This is consistent with results reported by Wong et al. (1993) [18] who saw a change in correlated activity near eye opening in the ferret. Epibatidine treated ferret s show a similar lack of correlation in their spontaneous activity at P25 [CI = 2.74 ± 0.09 (SEM; *n* = 770 units from 7 retinas)] at distances of 200μm (Fig. 2, F). Interestingly, the normal activity at P25, after eye-specific segregation is complete, lacked the correlated spatial activity present during ages when eye-specific inputs are segregating, and instead had similar CI values to ferrets where the cholinergic network was disrupted with epibatidine at P15 and P20.

### Epibatidine Treatment Prevents Eye-specific Segregation during Stage III Waves in the dLGN

**Fig 3:**
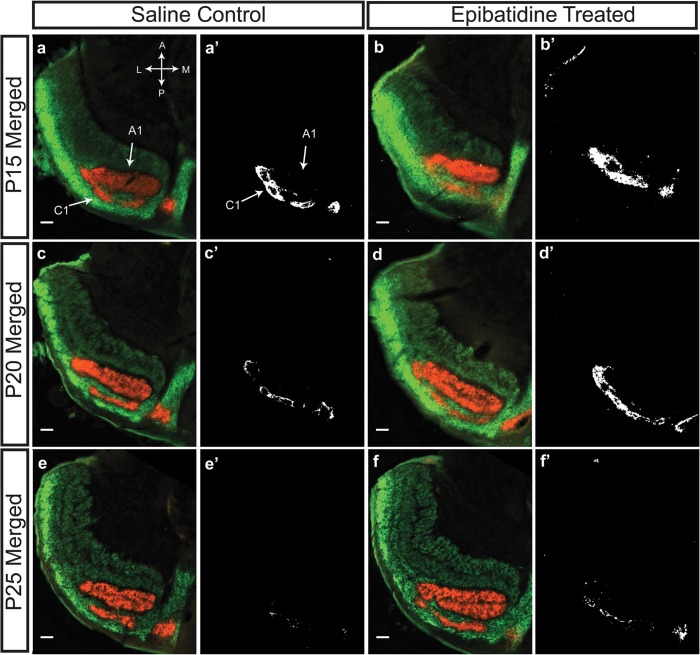
Epibatidine treatment prevents the segregation of the C/C1 lamina, but does not desegregate the already segregated A/A1 lamina. At P15 the contralateral and ipsilateral inputs to the C lamina have begun to segregate and the overlap between the two projections is reduced (A and A’). However, epibatidine treatment from P10-P15 prevents this segregation from occurring such that the majority of the C/C1 lamina remains overlapped (B and B’). At P20 the C/C1 lamina is almost completely segregated in saline treated ferrets (C and C’). Similar to earlier treatment, epibatidine from P15-P20 also prevents this segregation from progressing and the amount of overlap at P20 is similar to the amount observed at P15 in saline controls (D and D’). By P25 the last remnants of overlap between the C/C1 lamina in both saline and epibatidine treated ferrets is gone (E, E’, F, and F’). Scale bars are 200 μm. Overlap images were generated for signals 30% above background.

**Fig 4:**
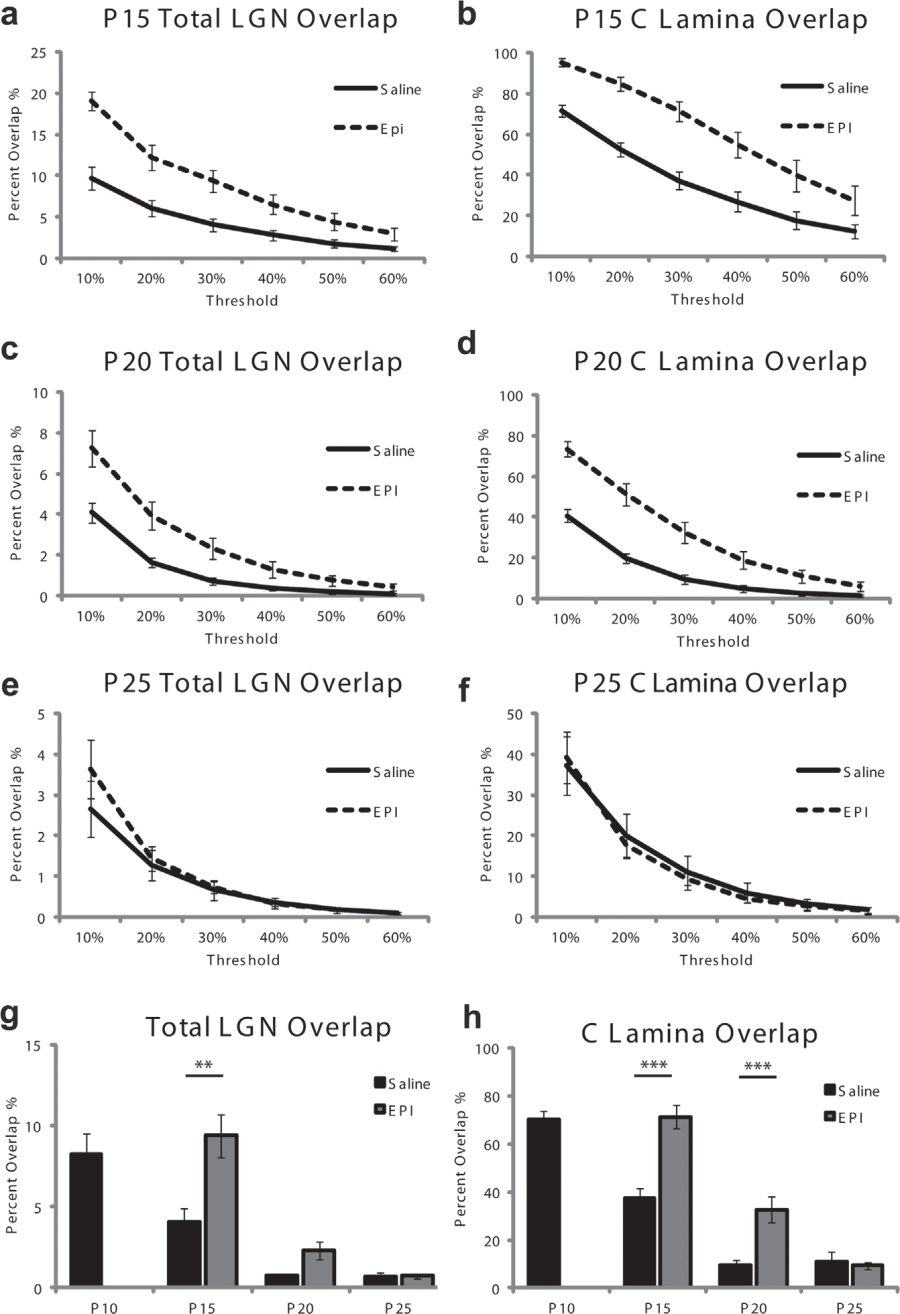
Difference in overlap between saline and epibatidine treated ferrets is not dependant on threshold. The percent of overlap measured decreases as a function of the threshold level. Epibatidine treatment (dashed line), as opposed to saline (solid line) from P10 to P15 increases the overlap at all threshold levels for both the total dLGN area (A) and just the C/C1 lamina (B). Epibatidine treatment from P15 to P20 also increases the amount of overlap observed, but only for threshold levels up to 40% (C and D). The overlap values at higher thresholds are near zero and cannot be distinguished between the two groups. There is no difference in the overlap at any threshold between ferrets treated with saline or epibatidine from P20 to P25 (E and F). Values of overlap between epibatidine (gray bars) and saline (black bars) treated ferrets are compared across ages at the 30% threshold cutoff for the total dLGN (G) and the C lamina (H). Time points reflect ages analyzed (i.e. P15 includes ferrets treated from P10 to P15). Data points reflect mean values ± SEM (** p <. 02, *** p <. 001).

Fig. 3 illustrates the progression of the segregation of the C/C1 lamina between P15 and P25 in the ferret. At P15 (Fig. 3, A, A’), saline treated ferrets have complete segregation of the A/A1 lamina, but overlap persists across the C/C1 lamina. The core of the ipsilateral C lamina has begun to segregate, but the border between the ipsilateral and contralateral projections is unrefined. Conversely, when treated with epibatidine from P10 to P15 (Fig. 3, B, B’), the C lamina retains the high degree of overlap observed at P10. A similar effect can be observed in ferrets treated from P15 to P20. At this age, saline treated ferrets (Fig. 4, C, C’) have a more segregated C/C1 lamina with only the very outer boundary of the ipsilateral C1 lamina retaining some overlapping projections with the contralateral C lamina. The epibatidine treated ferrets (Fig. 3, D, D’) are more overlapped and resemble the degree of maturation observed at P15. Finally, ferrets treated from P20 to P25 have no difference in their levels of overlap whether treated with saline (Fig. 3, E, E’) or epibatidine (Fig. 3, F, F’) suggesting that the cholinergic network is not necessary for the maintenance of overlap after P20.

To verify our results were not biased by our selection of a 30 percent threshold above background, we examined the percentage of overlap between ipsilateral and contralateral projections over a range of thresholds (Fig. 4, A-F). Large differences in overlap are observed at P15 and P20, but no difference is observed at P25. The effect is consistent across threshold values with the exception of the highest thresholds. At thresholds of 50 and 60 percent there is a floor effect that elicited no overlap in either saline or epibatidine conditions. Given that these high thresholds were not sensitive to the remaining overlap present in the C-lamina, we quantified the percentage of overlap from a threshold of 30 percent above background.

To test the consequence of epibatidine treatment on segregation after P10, we performed a mixed model ANOVA. There is a significant interaction between epibatidine treatment and the treatment age for both the total dLGN area (F(5,17) = 14.345; p < 0.0001), and the C/C1 lamina (F(5,17) = 33.267; p < 0.0001). We then conducted a post-hoc Tukey (HSD) test for multiple comparisons to identify which comparisons were significantly different. At P10 we observe 8.20 ± 1.31% (SEM) overlap between ipsilateral and contralateral inputs across the entire dLGN. When treated with saline for five days until P15 (*n* = 4) the observed overlap significantly decreases to 4.08 ± 0.80% (SEM) of the total dLGN area (p = 0.03) (Fig. 4, G). The projections from each eye onto the C/C1 lamina overlap across 37.16 ± 4.32% (SEM) of its area, significantly down from 69.90 ± 3.67% (SEM) at P10 (p<0.001) (Fig. 4, H). The total overlap in epibatidine treated ferrets at P15 is 9.38 ± 1.34% (SEM) of the total area, which is significantly more than the saline controls (p = 0.014), but not significantly different from the overlap at P10 [8.20 ± 1.31% (SEM), p = 0.947]. When we restrict our analysis to only the C/C1 lamina, 71.26 ± 4.88% (SEM) of the C/C1 area is overlapped in epibatidine treated ferrets, which is significantly more than in the saline controls (p<0.0001). There is no difference in C/C1 overlap between these ferret s and P10 [69.90 ± 3.67% (SEM), p = 0.99]. Importantly, we fail to see any desegregation of either the A/A1 lamina or the C/C1 lamina after epibatidine treatment, which suggests activity with disrupted correlations is sufficient for the maintenance of eye-specific inputs.

Overlap in saline controls (*n* = 5) is reduced at P20 to only 0.73 ± 0.17% (SEM) of the total dLGN area, which is a modest and not significant reduction in overlap from the start of saline treatment at P15 (4.08 ± 0.80% (SEM) total overlap, p = 0.084). However, the saline C/C1 lamina at P20 has overlap over 9.49 ± 2.20% (SEM) of its area which is significantly reduced from P15 C/C1 overlap (p = 0.001). At P20, epibatidine treated ferrets (*n* = 4) have 2.32 ± 0.53% (SEM) of their total dLGN area overlapped, which is not significantly greater than saline controls at P20 (p = 0.276). Once again, by restricting our analysis to the C/C1 overlap, we find a highly significant difference in overlap between saline and epibatidine treated [32.60 ± 5.32% (SEM); p < 0.0001]. The values of overlap for the epibatidine treated group at P20 are not significantly different from the saline group at P15 for total overlap (p = 0.742) or C/C1 overlap (p = 0.983). During this period of treatment epibatidine fails to desegregate the existent segregation of the C/C1 lamina, which is consistent with the results reported for P15.

At P25, the saline treated ferrets (*n* = 3) have only 0.67 ± 0.24% (SEM) overlap across the area of the dLGN. The C/C1 lamina is overlapped across 11.03 ± 4.13% (SEM) of its area. These values are consistent with the overlap observed at P20, suggesting eye-specific segregation has completed by that age. Epibatidine treatment from P20 to P25 (*n* = 3) does not produce a significant difference in the amount of total overlap [0.73 ± 0.15% (SEM), p = 0.109], or C/C1 lamina overlap [9.30 ± 0.71%, (SEM) p = 0.733]. Epibatidine treatment after P20 no longer disrupts the segregation of eye-specific inputs, nor does it desegregate the previously segregated inputs.

### Threshold Independent Analysis of dLGN Overlap

**Fig 5:**
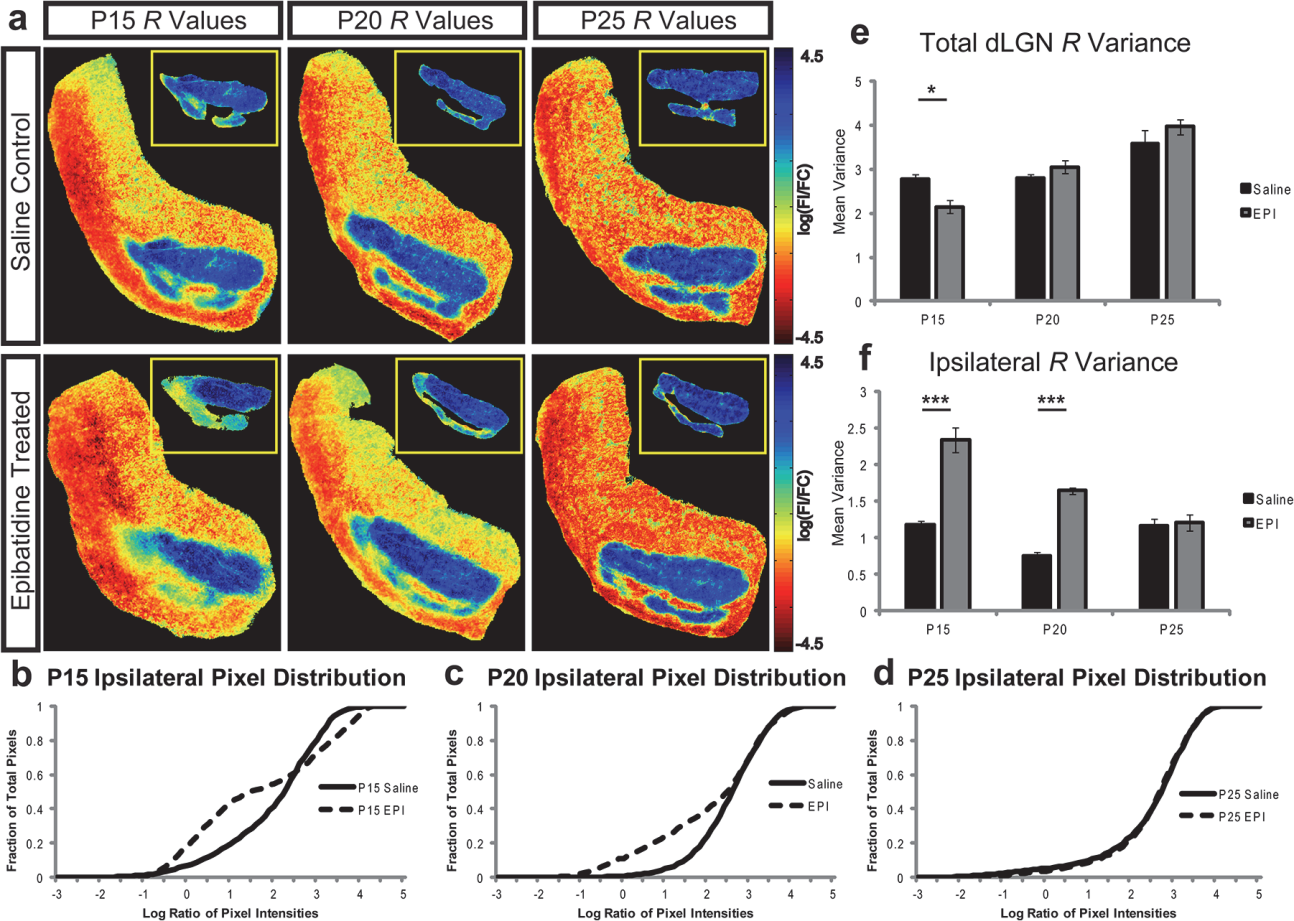
Threshold independent analysis of overlap confirms epibatidine prevents segregation during stage III waves. To avoid bias from selection of a threshold, we calculated the logarithm of the ratio of pixel intensities (*R)* between the ipsilateral and contralateral projections. (A) *R* values pseudo-colored and scaled from maximally contralateral (red) to maximally ipsilateral (blue) from saline and epibatidine treated ferrets from P10 to P15, P15 to P20, and P20 to P25. Pixels with similar contributions from each channel are colored yellow and green. Images are the same sections shown in Fig. 3. Insets are from an independent analysis where the ipsilateral projection was isolated and compared with an identical region in the contralateral image. The presence of non-blue pixels in the inset represents regions of more overlapped input. The cumulative fraction of pixels from the insets in A contain more ipsilateral only pixels (positive values) than overlapped pixels (near zero or negative pixels) in saline controls (solid line) compared to epibatidine treated (dashed line) at P15 (B) and P20 (C), but not P25 (D). Panel E shows the mean variance of the distribution of *R* values for the total dLGN across all ages. For the total dLGN, this technique is only sensitive enough to find a difference in overlap at P15. However, when we focus our analysis on only the ipsilateral projection (F) there is a highly significant difference at both P15 and P20. Note that in the ipsilateral only analysis, more variance is indicative of more overlap as the image contains the overlap of both ipsilateral and contralateral signals, as opposed to a distribution that has no overlap and only contains ipsilateral signals. Data values are mean ± SEM (* p<0.02; *** p<0.0001).

In an effort to rule out bias due to the selection of a threshold as the source of our results, we performed an analysis that does not require the arbitrary selection of a threshold for quantifying overlap. First proposed by Torborg and Feller [25] for dLGN image analysis, we calculated the log ratio of contralateral and ipsilateral signal intensities after a background subtraction for each pixel in the image (see Materials and Methods). The resulting value is negative for pixels dominated by contralateral projections, positive for pixels dominated by ipsilateral projections, and near zero if similarly occupied by both projections. Fig. 5 (A) illustrates the distribution of pseudo-colored contralateral and ipsilateral log ratio values (*R)* for the same images in Fig. 3. We calculated the variance of these *R* values to identify the relative degree of overlap between saline and epibatidine treated ferrets. In the full dLGN image, the greater the variance in *R* values, the less overlap, as more pixels will be maximally positive or negative. However, if there is greater overlap, there will be less variance due to a greater number of values in the center of the distribution. A mixed models ANOVA revealed a significant interaction [F(5,16) = 14.345; p<0.0001] between age and treatment condition when looking at the total dLGN overlap at P15 [Tukey (HSD) post hoc test, p = 0.014] but not at P20 (p = 0.276). Unfortunately this method of analysis is not spatially specific and our results were being overshadowed by the large variance in the texture of the contralateral A lamina relative to the small degree of overlap our threshold analysis revealed in the ipsilateral C1 lamina. To remove this noise we isolated the ipsilateral projection and calculated the ratio for what would only be ipsilateral pixels if there is no overlap between ipsilateral and contralateral projections (Fig. 5, A insets). We then measured the cumulative percentage of pixel values for the isolated ipsilateral images, (Fig. 5, B-D data from insets). Unlike the full dLGN projection, ipsilateral isolated images that are more segregated have tighter distributions due to mostly ipsilateral signals being present. Conversely, images that are less segregated have broader distributions of *R* values, as both ipsilateral and contralateral signals are present in the image. A mixed models ANOVA revealed a significant interaction between age and epibatidine treatment on ipsilateral pixel variance [F(5,16) = 33.267; p<0.0001]. A Tukey (HSD) post hoc test identified significant differences in the variance of ipsilateral pixel distributions between saline and epibatidine treated ferrets at P15 (p<0.0001), and P20 (p<0.0001), but no difference at P25 (p = 0.733) (Fig. 5, F), which is consistent with the results of our threshold analysis.

## Discussion

The ferret dLGN is divided into functionally distinct laminar structures. The largest laminar segregation is between the ipsilateral and contralateral eye-specific projections. Each eye-specific lamina is further subdivided into A/A1, which receives X and Y inputs contralaterally, and X input ipsilaterally, and C/C1 lamina, which receives and W and Y inputs contralaterally and W inputs ipsilaterally [26,27]. Within the A/A1 lamina, there is a further functional segregation between On and Off RGC projections [28]. Here we show that the C/C1 lamina, which differentiates from the A/A1 lamina between P5 and P10 [15] is mostly overlapped at P10. It has previously been reported that the maturation of Y and W cells occurs later than X cells [29], which is consistent with our result that the ipsilateral C1 lamina, which receives exclusively W cell input, segregates later than the A/A1 lamina. While the C/C1 lamina is still slightly overlapped at P25, it remains to be seen whether or not this is because the process is incomplete, or whether there simply remains some marginal overlap due to error in our measurement or incompleteness of the segregation process. There is some precedent for the latter, as the stratification between On and Off RGC arbors in the LGN is very tight for X ganglion cells, keeping them out of the intralaminar space. In contrast, Y and W ganglion cells do not tightly stratify and do bleed into the intralaminar space between On and Off layers [27]. Therefore, it is possible that the Y and W inputs to the C/C1 lamina boundary do not tightly adhere to their appropriate eye-specific layers and may always retain some marginal degree of overlap.

The previous view on retinal waves and eye-specific segregation was cholinergic stage II retinal waves are responsible for the segregation of eye-specific inputs and glutamatergic stage III retinal waves are responsible for the maintenance and refinement of RGC arbors in the dLGN. The properties of stage II waves and the mechanisms of their generation have been intensively studied [30–32]. Stage III retinal waves are driven by the release of glutamate from bipolar cells and propagate through the extra-synaptic spread of glutamate through the bipolar cell network [12]. When activity is blocked during stage III waves the eye-specific inputs desegregate [14], giving rise to the theory that stage III waves are important for the maintenance and refinement of RGC arbors. Epibatidine treatment during stage III waves, while able to disrupt the patterned activity, does not desegregate eye-specific inputs. Instead epibatidine treatment prevents the continuing developmental segregation of the C/C1 lamina between P10 and P20. Our results are not in conflict with experiments on the maintenance of eye-specific inputs because epibatidine does not block activity, but rather abolishes the instructive correlated patterns of activity inherent to retinal waves [6–9]. At P25 epibatidine treated ferrets do not have strong correlations present in retinal waves, but neither do the saline treated ferrets. It appears that normal patterned activity during stage III waves undergoes different sub-phases that may be related to the timing of the disassembly of the cholinergic network, the incorporation of light evoked visual responses around P20, or developmental changes in the bipolar circuitry that generates stage III waves [18]. These results combined suggest that any activity is sufficient for the maintenance of eye-specific inputs, but that normal patterned activity with local spatial correlations is necessary for the continuing segregation of eye-specific inputs.

**Fig 6:**
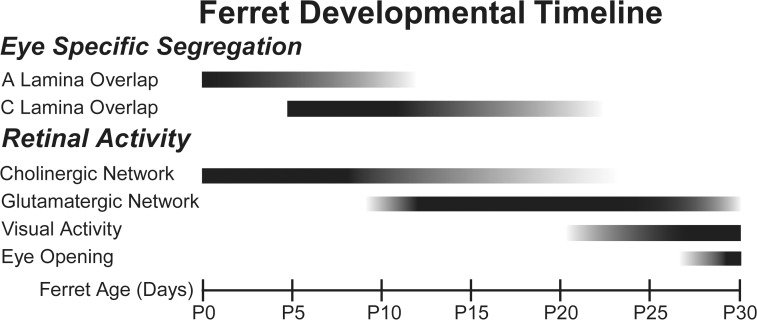
Eye-specific segregation persists through stage III waves, as does the influence of the cholinergic network on spontaneous retinal activity. A summary of the proposed developmental timeline illustrates the different timing of laminar segregation in the ferret dLGN, as well as the extended influence of the cholinergic network to coincide with the activation of the glutamatergic network during stage III waves. The C/C1 lamina becomes identifiable around P5, but remains highly overlapped at P10. Segregation is not complete until P25. The cholinergic network’s influence on retinal waves persists until at least P20 and is coincident with glutamatergic waves. For a comparative timeline of segregation and spontaneous activity in the mouse, see Ford and Feller (2012)[13].

Fig. 6 outlines our proposed developmental timeline for eye-specific segregation in the ferret dLGN. The A/A1 lamina of the ferret dLGN segregates between birth and P10, while the C/C1 lamina begins to differentiate between P5 and P10 and segregates between P10 and P25. The cholinergic network is directly responsible for spontaneous retinal activity between P0 and P10, and from our data either directly or indirectly contributes to the generation of spontaneous activity between P10 and P20. Once eye-specific segregation is nearly complete the influence of the cholinergic network is reduced. The glutamatergic network comes online at P10 and drives retinal activity either jointly or under the influence of the cholinergic network until eye opening around P30. The functional relevance of glutamatergic activity beyond the maintenance of segregated inputs is unknown, but may be important for the functional refinement of visual receptive fields or On and Off pathways [19,21,33,34]. The ferret retina becomes photoactive around P20, which may be related to the disassembly of the cholinergic network as a similar transition from stage II waves to visually evoked activity is observed in mice.

## Conclusions

Our study demonstrates that there are differences in the developmental time course of eye-specific segregation between the laminar structures of the ferret dLGN, such that the A/A1 lamina segregates first, followed by the C/C1 lamina later. This is in contrast to research on eye-specific segregation in the mouse which does not have laminar structures subdividing the dLGN. We demonstrate for the first time in the ferret that the effects of epibatidine on eye-specific segregation and spontaneous retinal activity can be seen as late as P20. Epibatidine’s effectiveness at disrupting the correlated structure of retinal activity corresponds to its effectiveness at preventing the segregation of eye-specific inputs. As opposed to experiments that block retinal activity, epibatidine treatment does not desegregate already segregated ganglion cell arbors. Instead, epibatidine prevents the continued segregation that would have occurred during normal development during that period of time. While our experiments cannot speak to differences in the role stage II and stage III waves play in the anatomical development of the dLGN, our work indicates that the distinction between stage II and stage III waves may be less clear than previously imagined. Further work needs to be done on the timing of the transition between cholinergic and glutamatergic retinal waves in the ferret. It is also necessary to explore the function of later stage III retinal waves on the refinement and organization of the ferret dLGN. We demonstrate here for the first time that eye-specific segregation continues to be disrupted by epibatidine treatment during stage III retinal waves in the ferret.

## Materials and Methods

### Ethics Statement

Timed pregnant and neonatal litters of fitch-coat ferrets were obtained from Marshall Farms (New Rose, N Y). All experimental procedures were performed in accordance with approved animal use protocols (protocol numbers 16676 and 16021) at the University of California, Davis. Ferrets were monitored for normal weight gain and growth throughout the course of the study [35], as well as for post operative distress from the experimental procedures.

### Retinal Wave Disruption

Surgical and drug injection procedures were similar to those described previously [5,17]. Mixed gender juvenile ferrets were anesthetized with inhalant isofluorane (Isothesia, Butler Schein, Dublin, OH), the eyelids were opened, and a small incision was made on the temporal side at the joining of the upper and lower eyelid. Intravitreal injections of either epibatidine HCl (1mM dissolved in sterile saline; Sigma, St. Louis, MO) or saline were administered daily for 5 days starting at P10, P15, or P20. The amount injected ranged from 2–4ul; 2.0 μl was injected on P10, and the amount increased by 0.25 μl for every other injected age, i.e. P12: 2.25μl, P14: 2.5μl, etc. Injections were administered into the vitreous humor of each eye using a 32 gauge needle attached to a Hamilton (Reno, NV) microsyringe. Injections were made slowly over the course of a couple minutes to reduce sudden changes in intraocular pressure. Eyes were treated with antibiotic ophthalmic gel (Vetericyn, Innovacyn Inc., Rialto, CA) after completion of the injection.

### Labeling of Retinogeniculate Afferents

Anatomical labeling of retinogeniculate afferents was performed as described previously [5,17]. On their fifth day of injections, ferrets received intravitreal injections of cholera toxin-β subunit (CTB) conjugated to fluorescein (488nm green label) in the left eye and CTB conjugated to rhodamine (594nm red label) in the right eye (2.5–4.0 μl depending on the age of the ferret; 0.5% in sterile saline; List Biochemical). Twenty-four hours later ferrets were euthanized with an overdose of pentobarbital (Fatal Plus, Vortech Pharmaceuticals, Dearborn, Michigan) via inter-peritoneal injection, transcardially perfused first with saline and then with 4% paraformaldehyde (Sigma, St Louis, MO). Brains were submerged in 30% sucrose solution and the thalamus was sectioned horizontally at 40μm on a freezing microtome. Sliced tissue was mounted on slides and cover-slipped with Vectishield (Vector Labs, Burlingame, CA).

### Image Analysis

All images were digitally acquired with a CCD camera (SPOT Diagnostic); photo exposure was automated. Raw images of the dLGN were imported to Photoshop (Adobe Systems, San Jose, CA) and converted to a grayscale image. Images were then thresholded and binarized at a range of signal intensities ranging from 20% to 60% above background (designated as a non-retino-recipient portion of the tissue slice medial to the dLGN; based on previous studies [17]. The dLGN area was then cropped to exclude the optic tract and medial intralaminar nucleus. Measurements of the area of ipsilateral C lamina and total dLGN were calculated by tracing the border of the intralaminar space from the cropped thresholded image (ImageJ; Wayne Rashband, NIH). Measurements of overlap were calculated by multiplying the thresholded ipsilateral eye inputs and thresholded contralateral eye inputs from the same dLGN tissue sample. After inversion, the resulting image contained black pixels at locations where pixels from the ipsilateral and contralateral signals were both present. These black pixels were counted for the quantification of overlap relative to the size of the dLGN. For measuring the C/C1 overlap the same method was used except the cropped ipsilateral eye input image containing only pixels in the C1 lamina was multiplied against the paired contralateral image. Four sequential sections through the middle 200–500 μm portion of the dLGN were analyzed for each ferret and averaged to give a single estimate of overlap. Data was excluded from ferrets in which the eye was damaged by the injections or retinogeniculate labeling appeared incomplete.

We conducted a separate threshold independent analysis to replicate and validate our threshold analysis [25]. For the same images used in the threshold analysis, background fluorescence was subtracted using a rolling ball filter of 200 pixels in ImageJ. Images were normalized to an 8-bit gray scale and the dLGN was cropped to exclude non-retino-recipient thalamic nuclei. We then calculated the logarithm of the ratio of signal intensities between the ipsilateral and contralateral projections:

$$R\;=\;{log}_{10}\left(\frac{F_{I}}{F_{C}}\right)$$

where *F_I_* is the fluorescence intensity in the ipsilateral channel and *F_C_* is the fluorescence intensity in the contralateral channel. Pixels that are dominated by contralateral input have negative *R* values while pixels that are dominated by ipsilateral input have positive values. Pixels that are overlapped between the two inputs have near zero values. We then measured the variance of the distribution of *R* values. Images with little overlap between the ipsilateral and contralateral projections have broader distributions and more variance than images with greater overlap, as more overlap results in pixels with values near the center of the distribution and fewer on the flanks. One drawback of this analysis is that it is not spatially specific and not sensitive to the relatively small changes in overlap localized to the C/C1 border. To address this, we isolated the ipsilateral projection and examined pixels in the same spatially specific region of the paired contralateral image. When the analysis is conducted this way, the distribution of values is entirely dominated by the ipsilateral projection. Smaller values of variance indicate that there is little overlap as the distribution has less contralateral signal. Greater values of variance indicate that more of the ipsilateral projection is overlapped with contralateral pixels.

### Multi-Electrode Array Recordings

MEA recordings were performed as previously described [8,36]. Ferrets were euthanized with a lethal dose of pentobarbital (0.1–0.2 ml) via inter-peritoneal injection. The eyes were enucleated and the retinas were removed and stored in buffered and oxygenated media (Eagle’s minimum essential medium [MEME], M7278; Sigma-Aldrich, St. Louis, MO) at room temperature. A piece of retina was placed ganglion side down onto a 60-channel MEA (Multi-Channel Systems, Tubingen, Germany), and held in place with a piece of dialysis membrane (Spectrapore 132130; Spectrum, Los Angeles, CA). The tissue was superfused with buffered MEME at 1–2 ml/min at 37°C. The array electrodes were 30μm in diameter and arranged in an 8 x 8 rectilinear grid with an interelectrode spacing of 200μm. At this distance the signal for a given cell appeared on only one electrode, so each cell isolated was assigned the spatial coordinates of the electrode that recorded its signal. Analog data was acquired at 20 kHz per channel simultaneously from each electrode. After the retina was set up on the MEA, the tissue was allowed to acclimate for 10–20 min. On the emergence of retinal wave events, recordings were performed for 10–15 min during which time overall firing rates appeared stable.

### Spike Identification

Raw data was digitally filtered with a 125-Hz high pass filter (four-pole Butterworth) sorting spike events. A threshold of 6SD was set for each channel and 1 ms of data before and 4 ms after a threshold-crossing event were stored for each negative-slope event. These candidate spike waveforms were then sorted with Offline Sorter (Plexon, Denton, TX) using the first three principal components of the spike waveforms. Coincident events within 0.5 ms of each other that occurred on all electrodes were attributed to perfusion noise and removed. Clusters were first identified using an EM cluster algorithm by Shoham et al. [37] then manually edited for clustering errors. Typically, the activity of one to three cells was recorded by each electrode.

### Correlation Analysis

The degree of correlated firing between pairs of recorded cells were calculated with cross-correlation functions and assigned a correlation index (CI). The CI measures the likelihood relative to chance that a pair of cells fired together within a given time window. The CI was computed as described by Wong et al. [18] using the following formula:

$$CI\;=\;\frac{\;Nab\left(-w,\;+w\right)\times \;T}{\left[Na\left(0,\;T\right)\times \;\left(2\;\times \;w\right)\right]}$$

where Nab(-w, + w) is the number of spike pairs from cells a and b for which cell b fires within w seconds of cell a, T is the duration of the recording in seconds, Na(0, T) and Nb (0, T) are the total number of spikes from cell a and b during the recording, and 2 × w is the width of the correlation window. Nab was computed using w = 0.1s and the cross-correlation function was binned at 0.005s.
